# Clinical Notes on Uterine Surgery

**Published:** 1886-04

**Authors:** 


CLINICAL NOTES ON UTERINE SURGERY. *By* J. MARION SIMS, A. B., M. D., *Late Surgeon to the Woman's Hospital, New York. Memorial Edition. Paper, pp. XI, 401. New York:* WILLIAM WOOD & CO. *1886. Chicago:* W. T. KEENER.

The merits and demerits of Sims's "Uterine Surgery" have been so fully and frequently discussed in so many American and foreign medical journals, from its first appearance in 1866 up to the present time, that the reviewer's task is a work of supererogation. It may not be amiss, however, to note that Bandl's recent anatomical investigations into the normal position of the non-gravid uterus sustain in all essential particulars the conclusions clearly and distinctly described by Sims so many years ago.

Messrs. William Wood & Company have conferred a boon upon the English-speaking members of the medical profession in placing at their disposal a cheap edition of a book absolutely essential to the specialist, and without which the library of the general practitioner would be incomplete. W. W. J.

